# Experimental and modelling studies of collision avoidance strategy choices and behavioural characteristics in interweaving pedestrian flow

**DOI:** 10.1098/rsos.220187

**Published:** 2022-07-13

**Authors:** Qiu Yun Luan, Shao Bo Liu, Zhi Jian Fu, Jie Yin Lyu

**Affiliations:** ^1^ Intelligent Transportation Systems Research Center, Wuhan University of Technology, 1178 Heping Avenue, Wuhan Hubei 430063, People's Republic of China; ^2^ School of Transportation and Logistics, Southwest Jiaotong University, High-Tech Zone West Park, Chengdu Sichuan 610031, People's Republic of China; ^3^ CIMC Intelligent Technology Co. Ltd, High-tech South Seven Road, Shenzhen 518057, People's Republic of China

**Keywords:** pedestrian flow dynamics, interweaving pedestrian flow, pedestrian flow experiment, collision avoidance, multi-nomial logit model, long-short-term-memory

## Abstract

The mechanisms of collision avoidance (CA) behaviours in interweaving pedestrian flow movements are important for pedestrian space planning and emergency management but not well understood yet. In this paper, a series of controlled interweaving pedestrian flow experiments with different densities are carried out to investigate the CA behaviours, especially CA strategy choices. Four types of CA strategies are manually identified in these experiments. Nine characteristic parameters based on the trajectory data are defined to explore the characteristics of CA behaviours. The experimental results reveal that (i) the CA behaviours change with density levels; (ii) heterogeneities can be found for individual pedestrians; (iii) the defined characteristic parameters show different statistical features for different types of CA strategies, and correlations exist between most of the parameter pairs; (iv) it usually takes 0.5–2.5 s to complete a CA process with a trajectory length of 0.5–3.5 m. A multi-nomial logit (MNL) model and a long-short-term-memory (LSTM) model are established respectively for predicting pedestrians' choices of CA strategies using the selected characteristic parameters as inputs. The modelling results prove the importance of using time-series data for pedestrian behaviour modelling, and the LSTM models show advantages over the MNL model at this point.

## 1. Introduction

In recent decades, with the rapid development of urbanization, safety and efficiency problems caused by large-scale activities, dense passenger flow and other crowded places have been paid more and more attention. These places usually have areas where pedestrian movement directions are complex and interweaved, such as atrium in shopping mall, interweaving areas in subway station, pedestrian crossings and public squares etc., which improves the complexity and difficulty of crowd management. However, the mechanism of pedestrians’ microscopic movement behaviours and decision makings during interweaving movement is still not clear so far, and the existing microscopic pedestrian flow simulation models also lack real data of pedestrian interweaving movement for the parameter calibration and model validation.

At present, there are mainly two streams of studies of pedestrian dynamics. One uses simulation models to predict and reproduce pedestrian behaviours. Another uses experiments and field investigations to obtain empirical data, so as to analyse and understand pedestrian behaviours.

Representative simulation models of pedestrian dynamics developed so far include cellular automata model, social force model, agent-based model and data-driven models such as neural networks. Most of these models are designed to simulate step-by-step pedestrian movement at microscopic level, in which collision avoidances (CAs) against other pedestrians or obstacles around have been involved. Examples of this stream of study include a cellular automata model of asymmetric simple exclusion process that was used to describe a situation where pedestrians walk through a crowded area to investigate how crowds may interfere with pedestrian behaviour [1]. The social force model has also been modified by introducing long-range conflict avoidance behaviours with the consideration of pedestrians' acceleration, deceleration or detour conflict avoidance strategies, and the authors concluded that conflict avoidances cannot be ignored for the interactions among pedestrians [2]. Existing simulation studies also tried to explored microscopic pedestrian movement interactions in even finer scale, e.g. Crociani *et al*. [3] presented an improved microscopic model aimed at the precision in the microscopic group dynamics, with particular attention to the shape of dyads. Considering the more and more rich data sources of pedestrian movement and the breakthroughs in machine learning, recent efforts also have tried data-driven methods such as neural networks, especially for pedestrian movement trajectory predictions, e.g. Huang *et al*. [4] proposed a spatial-temporal graph attention network, based on a sequence-to-sequence architecture to predict future trajectories of pedestrians, and adopt an extra long-short-term-memory (LSTM) network to encode the temporal correlations of interactions. Martin & Parisi [5] proposed a method based on generalized regression neural networks, which has been shown to be able to simulate the trajectory of a pedestrian avoiding an obstacle from any direction. However, most of the previous simulation studies have focused on the microscopic-level CA behaviours, i.e. the step-by-step locomotion of pedestrians. Microscopic models that consider long-range pedestrian CA behaviours, i.e. pedestrians decide a CA strategy such as accelerating, decelerating or detour before actually carrying out step-by-step locomotion, are still not very popular, and existing long-range CA behaviours models usually lack empirical data to validate the behaviours rules.

Besides simulation models, great efforts have also been put on empirical studies of pedestrian flow dynamics under various situations. There have already been many empirical studies on pedestrian dynamics by considering pedestrians’ age [6], gender [7], culture [8], visibility condition [9], density [10], angle [11,12], facility design [13], time-dependent information [14] and other factors. These studies usually involve several typical pedestrian flow scenarios, including uni-directional flow, bi-directional flow, multi-directional flow, intersecting flow, bottleneck etc. Examples on this direction may include: Seyfried *et al*. [15] investigated the single-line pedestrian flow characteristics through experiment and found the linear relation between the velocity and the inverse of the density in low–medium density conditions. Chen *et al*. [16] conducted an experimental study on the effect of three obstacle layouts (i.e. parallel, convex and concave layouts) on pedestrian flow in corridors. The T-junction and crossing flow experiments with different intersection angles [17] were also conducted, and the phenomenon named stripe formation [18] has been observed. Guo *et al*. [19] took the classroom as the experimental site for pedestrian evacuation studies considering pedestrians under normal and limited visual ability conditions, and the factors related with evacuation process such as capacity, pedestrian interactions and following behaviour were investigated. By now, a lot of studies have concentrated on bidirectional flows [20,21] where the intersecting angle of the two pedestrian flows is close to 180°. The fundamental diagrams of various flow types were compared and showed no apparent difference with respect to the intersecting angle 90° and 180° [22]. Bottleneck experiments were also carried out to explore the pedestrian behaviours and reactions in crowded situations [23,24]. The arching phenomenon and zipper effect are typical self-organized phenomena in the type of experiment [25]. However, although the existing empirical studies have already covered many typical pedestrian flow scenarios, investigations on interweaving flow where pedestrians move at any possible directions independently in open spaces are still rare. And there is also a lack of empirical data on long-range pedestrian CA behaviours under complex pedestrian flow environment, including the interweaving flow scenario.

Therefore, it can be concluded that CA is one of the key behavioural aspects in the study of pedestrian movement, especially the interweaving flow scenario where conflicts between pedestrians are usually more intense. Some of the previous studies have focused on the impact of obstacles on evacuation efficiency, such as placing obstacles in front of the exit to study the walking behaviour of pedestrians [26,27]. In this case, pedestrians usually change their movement strategies in advance to avoid collisions with obstacles. Other studies placed an object or a standing pedestrian as an obstacle, to explore the obstacle avoidance mechanism of pedestrians during movement [28,29]. These pedestrian obstacles avoidance studies mainly focused on how pedestrians change their direction, speed and the critical distance during CA, and showed that the change of movement direction was related to the angle and distance between obstacles and pedestrians. For example, Moussaïd *et al*. [28] proved that pedestrian movement direction change was related to the angle and distance with obstacles. Lv *et al*. [30] conducted CA experiments and found that most pedestrians change movement direction when they are 0.9–2.0 m away from obstacles. Parisi *et al*. [31] introduced the self-stopping mechanism in the social force model and proposed the private space concept, and defined that if there were obstacles or other pedestrians in the private space, the expected speed of individual pedestrians would become zero. In an experiment involving only two pedestrians with crossing trajectories, Paris *et al*. [32] extracted the two pedestrians' speed and movement direction to study the interactions between pedestrians, so as to predict the behaviour of the subject under potential collisions. Some studies treat standing pedestrians as obstacles and explore the critical distance, which is the distance from the obstacle when the pedestrian wants to start avoiding it. In the very low-density pedestrian movement experiment, when one pedestrian is not moving, the minimum avoidance distance is no more than 0.75 m; when both pedestrians are moving, the minimum avoidance distance is no more than 1 m; in the case of a potential perpendicular collision, and the minimum avoidance distance is no more than 0.68 m in the case of a head-on conflict situation [33]. The average critical walking space for avoiding standing pedestrians is 2 m^2^, and the average critical walking space for avoiding moving pedestrians on the opposite direction is about 2.64 m^2^ [34], and changes of the crossing angle also have impact on the avoidance distance [35]. Olivier *et al*. [36] investigated CA between two pedestrians by focusing on the conditions that lead to CA manoeuvres in trajectories; they concluded that pedestrians are able to accurately estimate their reciprocal distance at the time the collision will occur, and to mutually adapt this distance.

In general, the existing researches have mainly focused on the impact of fixed obstacles on moving pedestrian, and the CA mechanism of pedestrians facing fixed obstacles. However, there is still a lack of relevant research on pedestrians’ CA behaviours by seeing other pedestrians as ‘moving obstacles’, especially the complex long-range CA strategies in interweaving pedestrian flows where pedestrians from different directions do not have a fixed angle of interaction. The behavioural mechanisms and the characteristic parameters behind this phenomenon have not been well understood yet. Therefore, in order to further study the complex interactions between pedestrians in interweaving areas, this paper organized a series of interweaving pedestrian flow experiments in which pedestrians under different density levels walk from many different directions and cross each other in free angle. The focus of this study is mainly about observing and analysing of these behavioural strategies of pedestrians for CA facing ‘moving obstacles’ in the interweaving movement, including detour, acceleration, deceleration and stop strategies for CA. And the influence factors of pedestrians choosing the above four CA strategies are also empirically analysed. Based on the interweaving pedestrian flow experiment of CA strategies, this study reveals the dynamic characteristics of pedestrians' CA behaviours during walking across the interweaving area. A multi-nomial logit (MNL) model and a LSTM model are used to predict pedestrians’ choices of CA strategies. These results are useful for revealing the behavioural mechanisms of interweaving pedestrian flow movement, and for the simulation model development and parameter calibration of pedestrian flow micro-simulation models.

## 2. Experiment set-up

**Figure 1.**
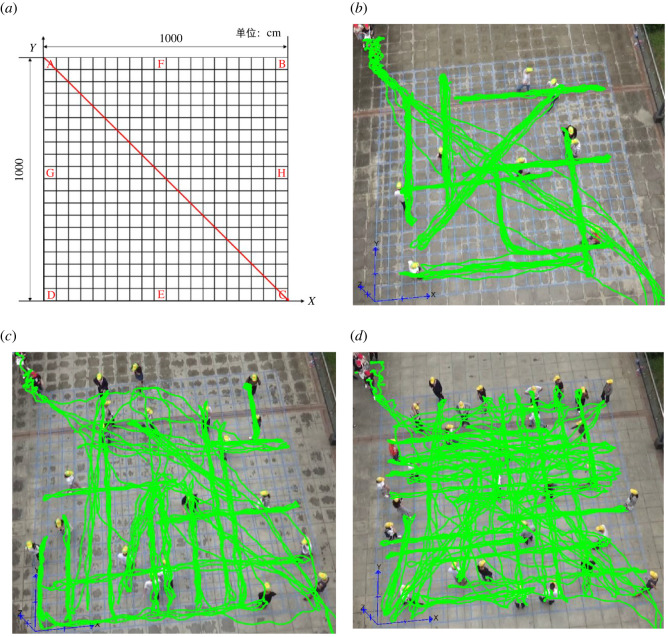
The layout of the experiment site and the superposition of the trajectories of all participants in the three groups of experiment with different density levels. (*a*) Layout of the experiment site and mark of key positions. (*b*) Trajectories of the participants in Group 1 of the experiment with 10 subject participants (SPs) and 9 passers-by persons (PPs). (*c*) Trajectories of the participants in Group 2 of the experiment with 10 SPs and 20 PPs. (*d*) Trajectories of the participants in Group 3 of the experiment with 10 SPs and 30 PPs.

The experiment was carried out in an outdoor square in Wuhan University of Technology. The experiment site was a 10 × 10 m square-shaped area which was marked by blue tapes on the ground and divided into uniform square grids to facilitate the experiment set-up. The length of each small square grid's side was 0.5 m. A high-definition video camera was placed at a height of 15.7 m above the site to record the experiment at 25 frames s^−1^. The entire experiment site was recorded in an approximate top-down manner to ensure that everyone's trajectory can be clearly seen and can be extracted by pedestrian trajectory recognition algorithms. A total of 40 students aged 18–23 years participated the experiment. For the convenience of video recognition and tracking, participants were asked to wear yellow or red hats. After the experiment, the PeTrack software was used to extract pedestrians' trajectories by identifying and tracking the coordinates of participants’ heads in the video records [37]. The PeTrack software has been widely used in pedestrian empirical studies for pedestrian movement trajectory data collection and data analyses. Then, the trajectory data extracted from this experiment were used to further analyse the pedestrian flow characteristics in this study. Figure 1 shows the layout of the experiment site and the superposition of trajectories of all participants.

This experiment has been designed for studying pedestrian CA behaviours in interweaving pedestrian flow area, in which pedestrians acted as obstacles to each other's movement. At the beginning of the experiment, participants were arranged in the site shown in figure 1 and were divided into two categories: (i) Participants wearing yellow hats acting as ‘passers-by persons’ (referred as ‘PPs’ hereafter) and (ii) subject participants (referred as ‘SPs’ hereafter) wearing red hats, whose CA behaviours would be the focus of this study. Three groups of experiments have been carried out (figure 1*b–d* and table 1), with 10 SPs in each group, but 9, 20 and 30 PPs in each group to simulate three different density levels. For each group of experiment, the 10 SPs are all the same 10 persons. The experiment was repeated three times for each different density level (table 1). The participants and their positions did not change in three times of repetitions. In order to minimize the influences of random factors as much as possible, the experimental results of the three times' repeats of each experiment group were averaged (where applicable) during the data analyses.

**Table 1.** Experiment set-up.

	Group 1 (low density)	Group 2 (medium density)	Group 3 (high density)
no. SPs	10	10	10
no. PPs	9	20	30
participant positions and trajectories	figure 1*b*	figure 1*c*	figure 1*d*
times repeated	3	3	3

At the beginning of each group of experiments, the PPs were asked to stand at the given positions as shown in figure 1, and each PP was required to choose a destination position randomly. Once the destination position was chosen, it should not be changed during one time of experiment. At the same time, 10 SPs were asked to queue outside the site around point A (figure 1), waiting for the starting command. Upon receiving the instruction to start the experiment, the SPs were asked to walk in their normal speed across the interweaving area from point A to point C as soon as possible based on their own walking habits. And at the same time, the PPs were asked to walk back and forth from their initial positions to their own destinations as soon as possible, and thus became ‘moving obstacles’ to SPs; the PPs were also required to walk at their normal speed based their own walking habits. Both PPs and SPs are free to choose their own way of avoiding other pedestrians (detour, decelerate, accelerate or stop and waiting for others to pass and then continue to move forward). There is no difference in the instructions given to SPs and PPs with regard to walking speed and CA. They are marked with red hats or yellow hats only for the easy of experimental organization and data processing purposes. Considering the complexity of the CA behaviours, if the CA behaviours of all the SPs and PPs are included in the study, the data acquisition and processing would become an unacceptable difficult task. Therefore, only the SPs' CA behavioural characteristics and strategies would be carefully analysed in this study, and the PPs are only acting as passers-by persons whose interactions with SPs would be considered but their CA behaviours would not be the focus in this study.

## 3. Analysis of the experimental results

Video records of the experiment were played frame by frame to manually identify the qualitative characteristics of CA strategies of each SP during each time of CA. And then the trajectory data of all the participants were used to study the quantitative characteristics of the CA strategies. This section presents the definition of the types of CA strategies manually identified, the characteristic parameters used for studying the CA behaviours and the analysis of the experimental results based on those definitions.

### 3.1. Definition of pedestrians' collision avoidance strategies and the segmentation of trajectories

**Figure 2.**
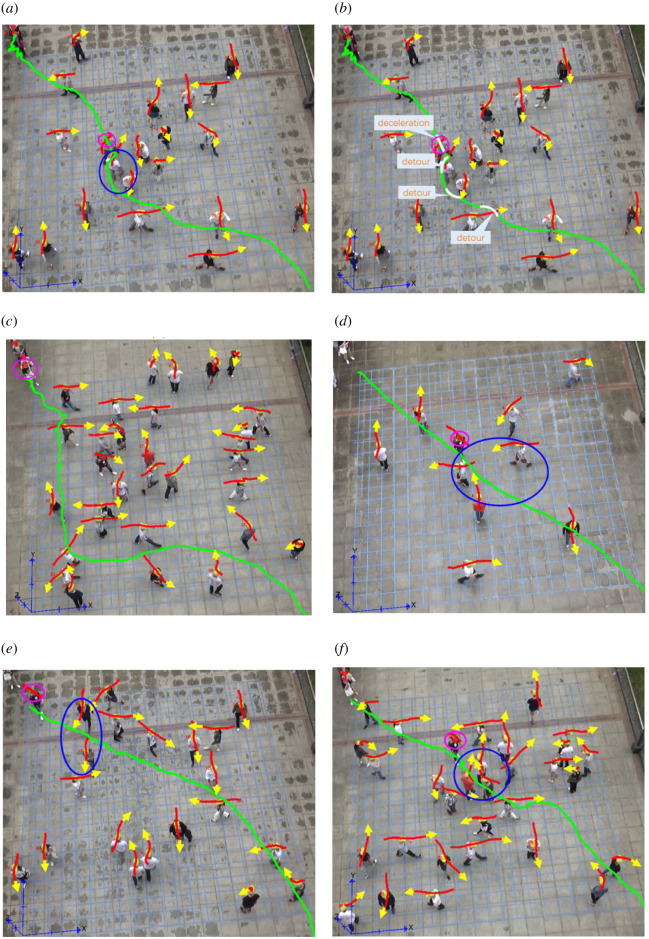
The screenshots and illustrations of the typical CA strategies in the experiments ((*a*), (*d*), (*e*) and (*f*) are the screenshots and illustrations of pedestrians adopting CA strategies of detour, acceleration, deceleration and stop, respectively, (*b*) shows the trajectory and the CA strategies adopted by the pedestrian in (*a*), and (*c*) shows the trajectory of a pedestrian adopted the detour behaviour along the boundary).

Adopting CA strategies in densely interweaving crowds is necessary to avoid conflicts with other pedestrians. By observing and analysing the pedestrian CA strategies in the three groups of experiments, four types of typical pedestrian CA strategies have been identified, including detour, acceleration, deceleration and stop (waiting). Examples of these typical CA strategies in the experiment have been illustrated in figure 2 using screenshots of the experimental videos and the trajectories extracted from the PeTrack software.

In figure 2, the pink circles indicate the SPs adopting the CA strategies, and the blue circles indicate the PPs in the region of interest that affect the SPs when they adopt the CA strategies. The red curves above each PP's head represent the trajectory of PPs in 15 frames before and 15 frames after the current frame. Therefore, the length of a red curve also indicates the speed of the corresponding PP. The longer the curve is, the faster the PP is moving in the 30 frames. The yellow arrow at the end of each red trajectory represents the movement direction of the PPs at the moment. The green curves are trajectories of SPs. Red trajectories are plotted by using the coordinates of PPs' heads, while green trajectories are plotted by using the coordinates of SPs' feet. Both red and green trajectories are plotted by the **‘**show trajectories’ feature in the PeTrack software.

The above-mentioned four types of typical CAs are defined as follows:

(1) CA by acceleration strategy: pedestrians choose obstacle avoidance strategies mainly by predicting the distance from other pedestrians around, the relative angle of movement direction with other pedestrians, the speed and other relative factors. Acceleration CA strategy is that pedestrians are willing to make greater efforts to avoid collisions with other pedestrians and adopt a strategy of quickly passing through the potential collision area by accelerating movement without obviously detouring (detouring was not identified by visual observations). If detour could be identified visually, it would be classified as a ‘detour’ CA strategy as defined in (4), disregarding whether the SP is accelerating or not at the same time. An example of the acceleration strategy is shown in figure 2*d*.
(2) CA by deceleration strategy: a pedestrian may face situations where potential collisions with other pedestrians within the visual range have been predicted but the distances to them are too close to avoid the collision by accelerating or detour. Under this circumstance, the pedestrian is either unwilling to make effort to accelerate or unable to detour or accelerate due to reasons such as limited movement space. Another choice for the pedestrian would be to decelerate or stop and wait for others to pass and then move forward. Generally, pedestrians will take deceleration behaviour before they stop, but deceleration behaviour would not necessarily lead to stop. Pedestrians may stop only when deceleration still cannot avoid collisions. But pedestrians may also detour during deceleration. In this study, if detour could be identified visually, it would be classified as a ‘detour’ CA strategy as defined in (4), disregarding whether the SP is decelerating or not at the same time. An example of CA by deceleration strategy is shown in figure 2*e*.
(3) CA by stop (waiting) strategy: as mentioned above, pedestrians may stop and wait until others walk past to avoid potential collisions. CA by stop is the most labour-saving way for pedestrians to avoid collisions. The available movement space for the pedestrian under this circumstance is usually more limited and the conflicts of the pedestrian with others are usually more intense compared with other situations. The waiting strategy can be seen as a special case of the deceleration strategy, but considering the obviously distinguishable characteristics of these two stages, this paper would treat them as two separate CA strategies. An example of CA by waiting strategy is shown in figure 2*f*.
(4) CA by detour strategy: pedestrians usually choose the shortest path to reach their destination which means the ideal path should be a straight line. But, in reality, the pedestrians need to detour to avoid collisions if pure acceleration, deceleration or stop is not the best option. Under this circumstance, the pedestrian needs to avoid potential collisions by obviously changing the movement direction (which could be clearly identified through visual observation). Figure 2*a–c* shows some typical detour behaviours of ‘SPs’ in the experiment.

According to the observation of the experimental videos, it can be found that SPs adopting detour strategies may also accelerate or decelerate during the detour. It can also be found that two of the SPs adopt the detour strategy by moving along the boundary of interweaving area, which can be seen as a special situation of detour (such as the one shown in figure 2*c*). Therefore, the detour strategy defined in this paper includes four types of situations: (i) detour without change of speed; (ii) detour with acceleration; (iii) detour with deceleration and (iv) detour along the boundary. Considering the sample size that could be obtained from the experimental data, as well as the complexity of the behaviour analysis, this paper would not distinguish these four types, i.e. they are all ‘detour’ strategies.

According to the above definitions, each trajectory of the SPs was segmented into several **‘**normal walking segments (NWS)’ and **‘**collision avoidance segments (CAS)’ based on manual observations. A NWS means that during this segment, the SP moves freely toward the target and does not receive any obvious interferences from others, while a CAS means that the SP is affected by others and takes one of the four CA strategies, and the execution of the strategy lasts until this CAS ends. Figure 2 illustrated the concept of trajectory segmentation and the definition of NWS and CAS.

### 3.2. Definition of characteristic parameters for collision avoidance behaviours

**Figure 3.**
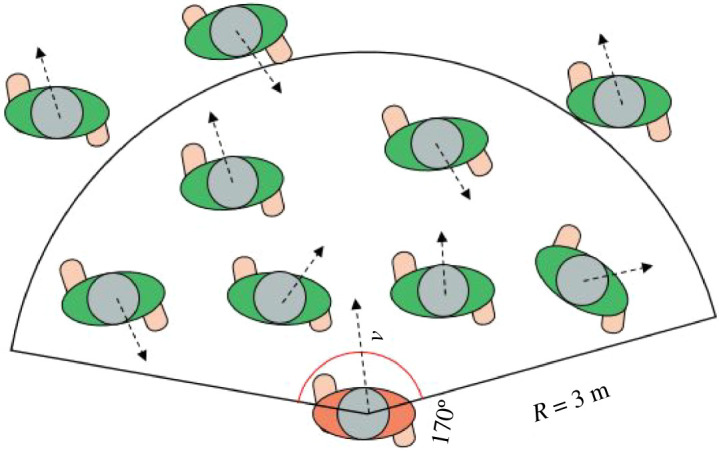
Illustration of the region of interest of a subject pedestrian.

As suggested in [38,39], movement behaviours of a pedestrian are usually considered to be primarily affected by the situations within his/her region of interest. The size of the region of interest could be conceptually given by the depth of decision-maker visual field. In order to distinguish and quantify the four CA strategies more objectively and accurately, this study assumes that the region of interest (visual field) in front of the pedestrian is a fan-shaped area with a centre angle of 170° and a radius of 3 m. Pedestrians within this range have the most important influences on pedestrians at the centre of the fan. The region of interest of a subject pedestrian is illustrated in figure 3.

**Figure 4.**
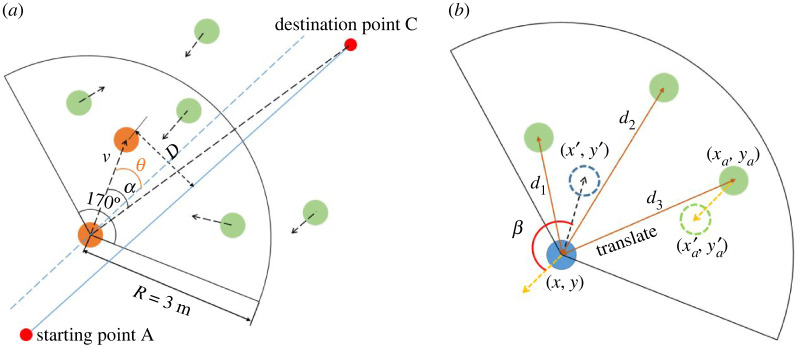
Illustration of characteristic parameters in pedestrian region of interest for quantifying CA behaviours.

In order to quantify and better study the behavioural characteristics of the four types of CA strategies, the following pedestrian behavioural characteristics parameters are defined in this paper to quantitative analysis and describe the CA behaviours of the SPs as shown in figure 4, tables 2 and 3.

**Table 2.** Definition of the pedestrian behavioural characteristic parameters.

*D*	offset distance from the centre line (metres, m)
*α*	the shifting angle of movement between the displacement vector of positions in two consecutive frames and the direction towards the destination (degrees, °)
*θ*	the angle deviated from the centre line (degrees, °)
$\bar{d}$	the average distance from the PPs in the region of interest (metres, m)
*d*_min_	the minimal distance from the PPs in the region of interest (metres, m)
$\bar{\beta }$	the average relative angle of movement directions to the PPs in the region of interest (degrees, °)
*β*_min_	the minimal relative angle of movement directions to the PPs in the region of interest (degrees, °)
*t*	the time to cross the interweaving area (seconds, s)
*L*	the length of the trajectory cross the interweaving area (metres, m)
*v_i_*_(*t*)_	the instantaneous velocity of the SP ***i*** at time ***t*** during crossing the interweaving area (metres per second, m s^−1^)

**Table 3.** Formulae used for calculating the pedestrian behavioural characteristic parameters.

*D*	$D=\left|\frac{x+y-1000}{\sqrt{2}}\right|$	(3.1)
*α*	$\cos\alpha =\frac{a\cdot b}{\left|a|\cdot |b\right|}=\frac{\left(x-x_{1})(1000-x_{1})(y-y_{1})(-y_{1}\right)}{\sqrt{{\left(x-x_{1}\right)}^{2}+{\left(y-y_{1}\right)}^{2}}+\sqrt{{\left(1000-x_{1}\right)}^{2}+{\left(-y_{1}\right)}^{2}}}$	(3.2)
*θ*	$\cos\theta =\frac{a\cdot c}{\left|a|\cdot |c\right|}=\frac{1000(x-x_{1})+1000(y-y_{1})}{\sqrt{{\left(x-x_{1}\right)}^{2}+{\left(y-y_{1}\right)}^{2}}+\sqrt{1000^{2}+1000^{2}}}$	(3.3)
$\bar{d}$	$d_{1}=\sqrt{{\left(x-x_{a}\right)}^{2}+{\left(y-y_{a}\right)}^{2}}\;\bar{d}=\overset{n}{\underset{i=1}{\sum }}d_{i}$	(3.4)
$\bar{\beta }$	$\cos\beta =\frac{c\cdot d}{\left|c|\cdot |d\right|}\;\bar{\beta }=\overset{n}{\underset{i=1}{\sum }}\beta_{i}\;\beta_{\min}=\min(\beta_{i})$	(3.5)
*t*	*t* = 0.04 × T (T is the frame rate of the experiment video, 25 frames per second)	(3.6)
*L*	$l=\sqrt{{\left(dx\right)}^{2}+{\left(dy\right)}^{2}}\;L=\overset{n}{\underset{i=1}{\sum }}l_{i}$	(3.7)
*v_i_*_(*t*)_	$v_{i(t)}=\frac{\left|l_{\left(t+1\right)}-l_{\left(t\right)}\right|}{\Delta t}$	(3.8)

A coordinate system is established as shown in the schematic layout and markup illustration of the experiment site in figure 1. The ‘centre line’ is defined as the line from the SPs' starting point A to their destination point C, as shown in figure 1 as well as in figure 4. The expression of the line AC is *y* = −*x* + 1000. Assuming that the coordinate of the SP is (*x*, *y*) and one of the PPs is (*x_a_*, *y_a_*) at the current frame, the coordinate of the SP in the last frame is (*x*_1_, *y*_1_). And the coordinates of the SP and the PP change to (*x*^′^, *y*^′^) and ($x_{a}^{\prime },\;y_{a}^{\prime })$, respectively, in the next frame. Then, the displacement vector formed by the last frame and the current frame is $a=(x-x_{1},\;y-y_{1})$, and the direction vector formed by the position of last frame and the destination is$\;b=(1000-x_{1},-y_{1})$. The vectors of the movement direction of the SP and the PP in the two frames are $c=(x^{\prime }-x,\;y^{\prime }-y)\;$ and $d=({x^{\prime }}_{a}-x_{a},\;{y^{\prime }}_{a}-y_{a})$, respectively.

### 3.3. Experiment data analysis

As mentioned above, the CAs of each pedestrian have been identified manually and the trajectory of each pedestrian has been divided into segments according to the four types of CA strategies. Each frame of trajectory data (including those variables defined above) was given a label denoting whether the pedestrian is at NWS or at CAS. If it is the second case, then which type of CA strategy the pedestrian is undertaking at this frame is also labelled. Based on this dataset, the CA behaviours of pedestrians can be analysed in detail, including the heterogeneous behavioural characteristics between different pedestrians as well as the characteristics and mechanisms behind the decision making during different types of CA.

#### 3.3.1. Heterogeneities in pedestrian collision avoidance behaviours

**Figure 5.**
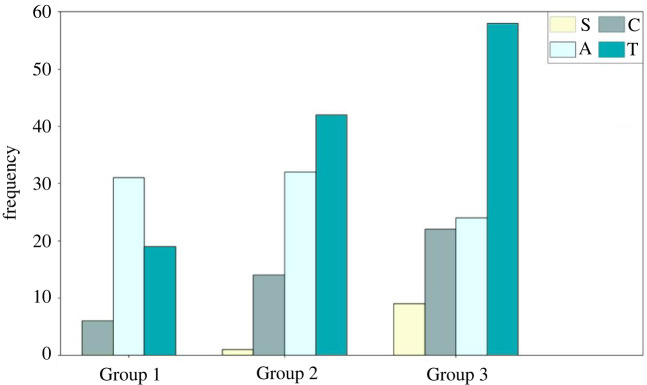
The total number of times of each type of strategy (frequencies) adopted in each experiment group.

**Figure 6.**
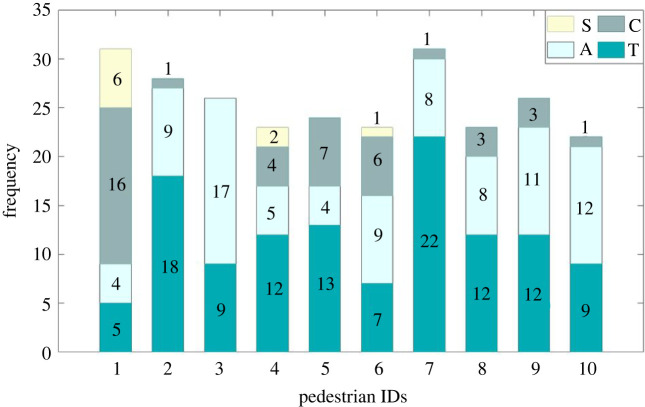
The total number of times of each type of strategy (frequencies) adopted by each SP.

The number of each type of CAS for each SP in the three groups of experiments is counted. The details are shown in table 4 and the aggregated results for each group of experiments or each SP are shown in figures 5 and 6. For convenience of description, the four types of CA strategies defined above are denoted as ‘A’ (Acceleration), ‘C’ (deCeleration), ‘T’ (deTour) and ‘S’ (Stop).

**Table 4.** Number of times of CAs of each SP in the three groups of experiments. Note: ‘A’, Acceleration; ‘C’, deCeleration; ‘T’, deTour; ‘S’, Stop.

pedestrian IDs and gender	Group 1	Group 2	Group 3
1. (woman)	2A/4C/1T	2A/5C/1T/1S	7C/3T/6S
2. (woman)	2A/3T	4A/1C/5T	3A/10T
3. (woman)	3A/1T	8A/3T	6A/5T
4. (man)	3A/2T	1A/2C/6T	1A/2C/4T/2S
5. (man)	1A/3T	2A/2C/7T	1A/5C/3T
6. (man)	6A/1C	2A/3C/2T	1A/2C/5T/1S
7. (man)	3A/3T	4A/6T	1A/1C/13T
8. (man)	3A	3A/1C/4T	2A/2C/8T
9. (man)	2A/1C/4T	3A/6T	6A/2C/2T
10. (woman)	6A/2T	3A/2T	3A/1C/5T

There are totally 258 segments in all of the experiments, among which the number of T segments is the biggest (46.1% of the total number of segments) and the number of S segments is the smallest (3.9% of the total number of segments).

As shown in figure 5, as the density of the interweaving area increases, the frequencies of T, C and S strategies increase apparently. The frequency of A strategy does not change obviously but its proportion decreases from 55.4% (in Group 1) to 21.2% (in Group 3). This is consistent with common sense that low density means less moving pedestrian obstacles to SPs, so the SPs are usually able to choose the shortest path to reach the destination by using acceleration strategy rather than unnecessary detours or waiting. As the density of interweaving area increases, the average distance $\bar{d}$ between pedestrians becomes smaller, and conflicts with PPs become stronger. In these cases, pedestrians may not be able to avoid collisions by merely decelerating, but have to stop or detour more frequently. Therefore, the frequencies of T, C and S strategies adopted in Group 3 experiment are apparently larger than other groups.

It can be seen from figure 6 that the frequency of the SPs taking each type of strategy shows apparent heterogeneous characteristics. For example, SP no. 1 tends to take C and S strategies more often; SP no. 3 takes more A strategies; SPs nos. 2 and 7 prefer T strategies. The reasons behind these heterogeneous characteristics of taking different types of CA strategies can be partially explained by figure 8, where the average of velocities of SPs under different statuses is compared.

**Figure 7.**
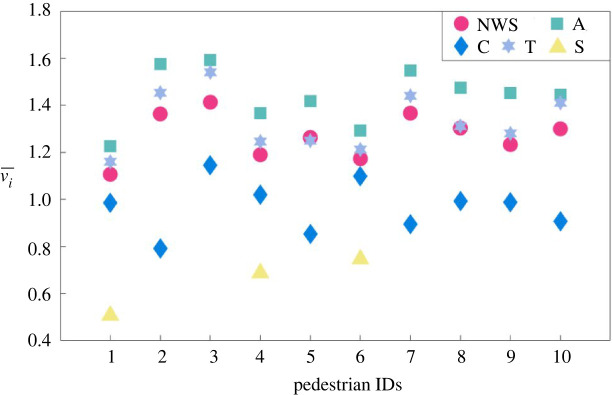
The average of instantaneous velocity of the SP ***i*** ($\bar{v_{i}})$ during ‘CAS’ of A, C, T, S strategies and ‘NWS’.

**Figure 8.**
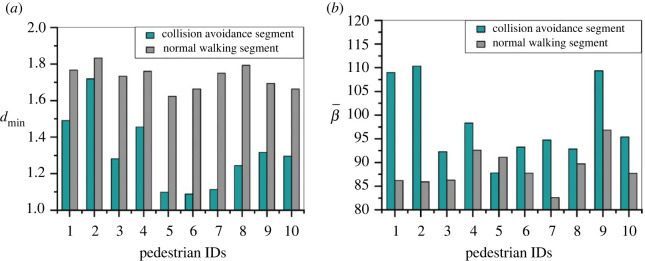
Comparison of the averages of characteristic parameters *d*_min_ and $\bar{\beta }$ for each SP during CAS and NWS.

Figure 7 reveals that the average movement velocities of SPs are apparently different not only during CAS and different types of NWSs, but also for each SP. The average velocities during CASs with T strategy is near to that of A strategy and is apparently higher than the other two types. This is due to the reason that SPs who carry out a T strategy may also accelerate or decelerate during the detour, but more acceleration cases have been observed in the video records of the experiment than deceleration. Besides, putting figure 7 and figure 8 together, it can be found that SPs with different normal walking velocities have chosen different CA strategies, indicating the potential influences of normal walking velocity on SPs' CA behaviours. For example, SPs nos. 1, 4 and 6 have relatively smaller normal walking velocities, and they are the only three SPs who carried out S strategy. SPs nos. 2, 3 and 7 have higher normal walking velocities and they carried out relatively more times of A or T strategies.

Besides walking velocity, other CA characteristic parameters may also have relationships with the CA strategy choices. Figure 8 shows the averages of *d*_min_ and $\bar{\beta }$ for each SP during CAS and NWS. Apparently, *d*_min_ during CAS is smaller than that during NWS, and $\bar{\beta }$ during CAS is bigger than during NWS, indicating closer interaction distances and more aggressive movement direction conflicts during CAs. Figure 8 also reveals the heterogeneity of SPs' CA strategy choices. Although the averages of *d*_min_ and $\bar{\beta }$ take relatively wide ranges during CASs (*d*_min_ ∈ (0.5, 1.7) m, $\bar{\beta }\in (92-110)\;\;\mathrm{degrees}$), the values of each SP differ from each other. For example, SPs nos. 1, 2, 4 and 9 have larger *d*_min_ and $\bar{\beta }$ during CAS than others, indicating that these pedestrians have higher motivation to take CA strategies in advance, that is when the distance to potential collision is still relatively long (*d*_min_ > 1.2 m) but if the relative movement direction implies high possibility of collision ($\bar{\beta }>100\;\;\mathrm{degrees}$), the SP may carry out collision avoiding. And this is different from other SPs such as SPs nos. 5, 6 and 7 whose behavioural characteristics are the opposite, that is they do not carry out collision avoiding until the potential collision distance is very near (*d*_min_ < 0.5 m).

Analyses of other characteristic parameters also yield similar conclusions. This implies the potential relationships between these parameters and the CA strategy choices, and the possibility of formulating or quantifying these relationships by establishing a forecasting model of CA strategy choices based on these parameters.

#### 3.3.2. Analysis of pedestrian collision avoidance strategies

##### 3.3.2.1. Overall characteristics of the three groups of experiment

**Figure 9.**
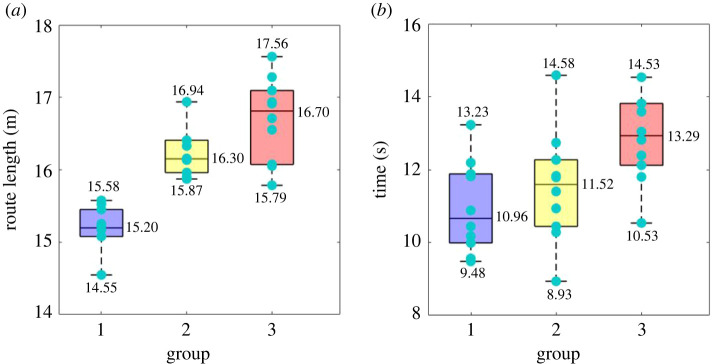
Basic statistics of macroscopic movement parameters of SPs during the three groups of experiments, including the length of each SP's route (*a*) and the travel time (*b*) of each SP.

**Figure 10.**
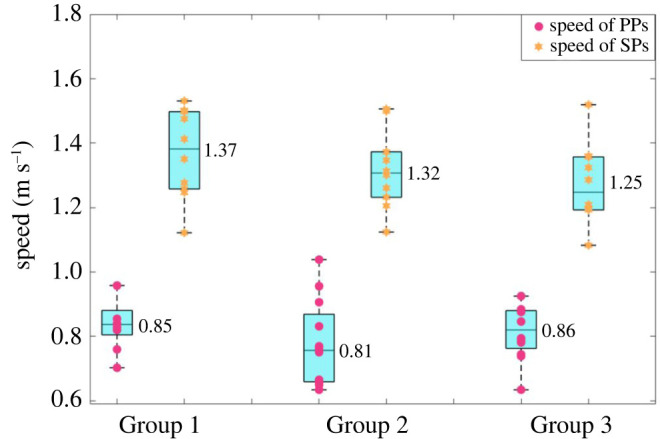
The average movement speed of each SP and PP.

**Figure 11.**
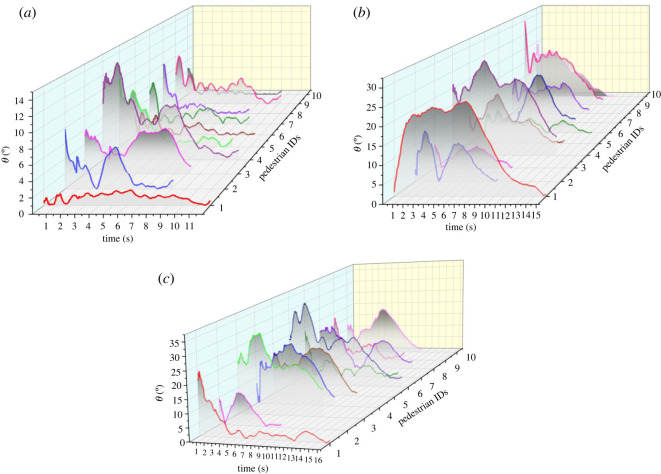
The angle deviated from the centre line (*θ*) against time in the three groups of experiments. (*a*) Group 1. (*b*) Group 2. (*c*) Group 3.

It can be found from figures 9 and 10 that as the density of the interweaving area increases from Group 1 to 3, the SPs have to spend more time to reach the destination, the average speeds decrease while the length of the trajectories increase, despite that the actual distance from the origin to the destination in the three groups of experiments had been always the same. This is obviously due to increasing conflicts of movement between pedestrians from Group 1 to 3 and can also be supported by the data shown in figure 11. Figure 10 shows that the average speed of PPs is smaller than that of SPs, and the average speed of PPs did not change much with the density level increases. As mentioned in §2 of this paper, the instructions given to SPs and PPs with regard to movement speed and CA behaviours were the same, i.e. they were all asked to walk normally towards their own destinations based on their own walking habits and there should be no differences between them with regard to the nature of CA behaviours. However, the paths of PPs are shorter than those of SPs, and the PPs were asked to move back and forth continuously, during which they had to make two ‘U-turns’ in each cycle of the walking path. Those PPs have to slow down to make each U-turn. These are the reasons that the average speed of PPs is smaller than that of SPs. And the average speed of PPs as well as the overall density levels (maximum density level is 0.31 person m^−2^ in Group 3) were small enough that the speeds of PPs have not been obviously affected by the increasing density levels and the conflicts between pedestrians.

Figure 11 shows the change of each SP's movement angle deviated from the centre line with time. *θ* indicates the extent to which the SPs change movement direction in avoiding obstacles. It can be seen that *θ* increases as the density of the interweaving area increases. In the low-density cases, *θ* is mostly between 0 and 8 degrees and the time periods with angle deviations usually range from 1 to 3 s. These two ranges are 0–20 degrees and 1–8 s for the medium-density cases, 0–30 degrees and 2–10 s for the high-density cases. Besides, it can be seen from the curves in figure 11 that the amplitudes and frequencies of fluctuations of these curves increase from Group 1 to Group 3. This indicates that with the increase of density, pedestrians would have to deviate from the shortest path more often and with larger deviation angles. And this in turn leads to more detours.

##### 3.3.2.2. Statistical characteristics of the collision avoidance segments

As mentioned above, the CAs of each pedestrian have been identified manually and the trajectory of each pedestrian has been divided into segments according to the four types of CA strategies. Here, the time (number of frames) between each pair of consecutive CASs is called an ‘interval’. It is found that the time lengths of the intervals widely distributed in the range of zero frame to over 100 frames, but the mean of the interval time length is 26.87 frames, and the 30% percentile is 10 frames. That is to say, despite the large standard deviation, most of the interval lengths (over 70%) are longer than 10 frames. This information could be useful for determining an appropriate time period for time-series analysis or modelling of the CA behaviours.

**Figure 12.**
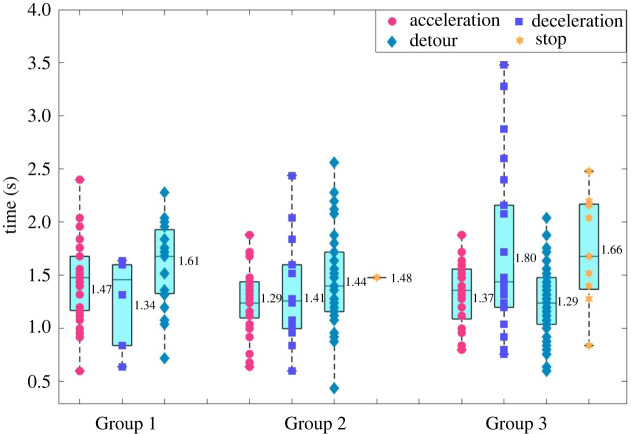
Boxplot of the time lengths of the four types of CASs in the three groups of experiment, numerical texts aside the box represent the means.

**Figure 13.**
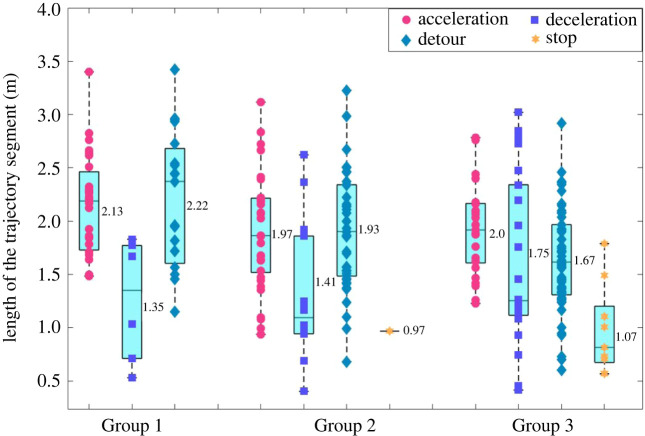
Boxplot of the lengths of the trajectories of the four types of CASs in the three groups of experiment, numerical texts aside the box represent the means.

Figures 12 and 13 present basic statistics of the time lengths of the CASs as well as the lengths of the trajectories of the CASs. These two figures intend to answer the following questions: (i) how much time does a pedestrian usually take to complete a CA? (ii) how long distance does a pedestrian need to complete a CA? (iii) are there differences between pedestrian flow conditions (e.g. density levels) or types of CASs?

It can be seen from figure 12 that the time lengths of the CASs range from 0.5 to 3.5 s, but most of them are within the range of (0.5, 2.5) s, with the mean of 1.3–1.8 s. No obvious differences could be identified either between different groups of experiments or different types of CASs. Figure 13 reveals that the trajectory lengths of CASs of A, C and T strategies range from 0.5 to 3.5 m, with the mean of 1.35–2.22 m. The trajectories of the S strategies are unsurprisingly the smallest, since pedestrians stop movement for a short time period in these situations. And the trajectories of CASs under C strategy is shorter than those under A and T strategies.

### 3.4. Analysis of characteristic parameters

Theoretically, pedestrians make CA decisions based on the perceived surrounding situations which could be partially defined by the characteristic parameters of CAs. It is therefore important to know whether these parameters are correlated with each other or not, and what the features of these parameters are under different types of CA strategies.

In order to test the correlations between these characteristic parameters, the maximal information coefficient (MIC) method has been used for each pair of parameters. MIC is a robust correlation analysis method proposed in 2011, featured as a maximization process of mutual information entropy between variables, and is proved superior to traditional correlation analysis methods such as Pearson or Spearman which is only applicable to linear correlation relationships [40].

**Figure 14.**
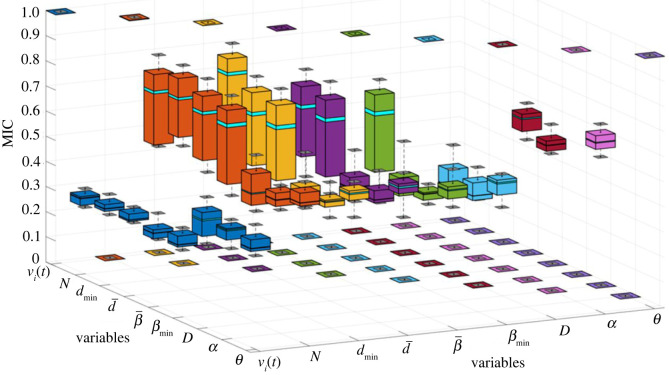
Boxplot of the averages of the MIC values between each pair of characteristic parameters for all the SPs.

The experiment data of the nine parameters defined in §3.2 are firstly organized by each SP ID of each experiment, and for each SP in each experiment, MIC process is run for each pair of parameters. The result for each SP in each experiment is a 9 × 9 table showing the MIC values of each pair of parameters. Then, the tables of all SPs are averaged and the averaged MIC values for each pair of parameters are shown in table 5. the corresponding boxplots of these MIC values are shown in figure 14. It can be seen from table 5 and figure 14 that, medium level of correlations (MIC greater than 0.4) do exist between most of the parameter pairs, among which N, *d*_min_, $\bar{d}$ and $\bar{\beta }$ have a correlation with most of the other parameters with MIC greater than 0.65.

**Table 5.** Averages of the MIC values between each pair of characteristic parameters for all the SPs.

	*v_i_*_(*t*)_	*N*	*d*_min_	$\bar{d}$	$\bar{\beta }$	*β*_min_	*D*	*α*	*θ*
*v_i_*_(*t*)_	1								
*N*	0.30	1							
*d*_min_	0.32	0.72	1						
$\bar{d}$	0.32	0.75	0.80	1					
$\bar{\beta }$	0.30	0.72	0.69	0.70	1				
*β*_min_	0.31	0.69	0.68	0.69	0.68	1			
*D*	0.43	0.54	0.52	0.52	0.50	0.49	1		
*α*	0.43	0.54	0.51	0.52	0.49	0.49	0.75	1	
*θ*	0.43	0.59	0.58	0.58	0.55	0.55	0.70	0.68	1

Table 6 presents the averages of characteristic parameters during the four types of CASs in each group of experiment. It can be seen that *v_i_*_(*t*)_ for A strategy is always the biggest in the four types of strategies, followed by T strategy; *α* for T strategy is always the biggest in the four types, followed by A strategy. As the density level increases (Group 1–Group 3), *v_i_*_(*t*)_ and *d*_min_ for A, C and T strategies decrease, indicating more and more intense interactions between pedestrians, and at the same time *D* and *α* for these three strategies increase, which can be accounted for by the increasing times of T strategies as well as the increasing interactions.

**Table 6.** Averages of characteristic parameters during CASs in each group of experiment.

group	strategies	*v_i_*_(*t*)_ (m s^−1^)	*D* (m)	*α* (°)	*d_*min*_* (m)
1	acceleration	1.49	0.54	10.86	1.59
	deceleration	0.98	0.17	4.87	1.59
	detour	1.42	0.48	11.08	1.31
2	acceleration	1.47	0.88	15.51	1.53
	deceleration	0.96	0.66	14.42	1.26
	detour	1.34	1.09	21.22	1.22
	stop	0.47	3.32	50.40	1.24
3	acceleration	1.36	1.12	21.41	1.23
	deceleration	0.93	0.43	11.76	1.20
	detour	1.30	1.24	23.37	1.34
	stop	0.59	0.61	12.93	1.16

**Figure 15.**
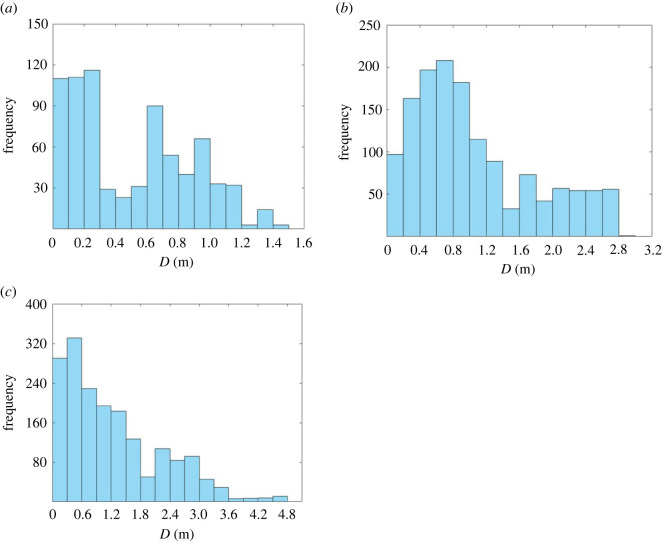
Frequency histogram of the offset distance from the centre line in the three groups of experiments when SPs choose the detour strategy. (*a*) Frequency of the offset distances from the centre line in Group 1. (*b*) Frequency of the offset distances from the centre line in Group 2. (*c*) Frequency of the offset distances from the centre line in Group 3.

Figure 15 shows the frequency histogram of the offset distance *D* from the centre line in the three groups of experiments when SPs choose the detour strategy. It can be seen that in the low-density situation (Group 1), the offset distances *D* are mainly between 0 and 0.3 m and 0.6–1 m (accounting for 73.95% of all the values). This range is 0.2–1.2 m (accounting for 74.86% of all the values) for medium-density situation (Group 2), and 0–1.8 m (accounting for 80.36%) for high-density situation (Group 3). Besides, the maximum value of *D* increases from 2.4 m in Group 1 to 3.7 m in Group 2 and 4.7 m in Group 3. This indicates that with the increase of density level, it becomes more difficult for pedestrians to avoid each other, and they need to detour longer distance to execute the detour strategy.

## 4. The collision avoidance strategy choice model

This section intends to further quantify the relationships between the CA strategy choices and those characteristic parameters discussed above, and then establish a model for predicting pedestrians' choices of CA strategies for interweaving pedestrian flow situations.

In pedestrian flow studies, pedestrians are usually assumed to make decisions of CAs according to potential collisions perceived within the so-called ‘region of interest’ or ‘visual perception field’ [41]. Existing researches usually assume there is an instantaneous mapping relationship between the perceived potential collision situation and the decision to be made, that is, using the parameters at the time point *t* − 1 to predict the action to be taken at time *t*. However, considering the nature fact that humans usually need at least 0.15–0.22 s [42] to respond to a visual stimulus, and in pedestrian travel circumstances, the decision-making time follows a distribution with the mean of 0.4 s [43], there are sufficient reasons to modify the time span of the above said mapping relationship from ‘instantaneous mode’ *t* to ‘time-series’ mode Δ*t*, where Δ*t* is a small time period during which pedestrians respond to potential collisions and make CA decisions. According to the analyses of the time intervals between CASs in this experiment in §3.3.2.2, this paper takes Δ*t* = 0.4 s for the following modelling studies. Specifically, this paper uses the data of characteristic parameters during the past Δ*t* = 0.4 s (10 frames in the experiment videos) as the inputs to build models for predicting pedestrians' CA strategy choices at the time step *t*. In order to test different types of models and compare the effect of changing ‘instantaneous mode’ to ‘time-series’ mode, a MNL model and a LSTM model are established, respectively.

### 4.1. The multi-nomial logit model for collision avoidance strategy choice

The process of a SP choosing a CA strategy can be seen as a complex decision-making problem. Decision makers usually choose the most beneficial option according to their own situation. The MNL model is widely used to describe the decision process of choosing from a series of alternatives based on the random utility theory. In this paper, a MNL model is established to predict the decisions of pedestrians on CA strategies in complex pedestrian interweaving area. The random utility method is used to estimate the utility function of each strategy and the strategy with higher utility would have a higher possibility to be chosen. Meanwhile, the effect of the main influencing factors can be quantitatively explored.

According to the random utility theory, the utility that quantifies a decision maker of choosing an option within a range of alternatives is regarded as a random function with two parts: a fixed term function and a random term function, then utility *U_ij_* of decision maker *i* choosing the alternative *j* can be expressed as follows:

4.1 $$U_{ij}=V_{ij}+\varepsilon_{ij},$$

where *V_ij_* is the fixed term part of pedestrian *i* choosing strategy *j*, and ε*_ij_* is the random term part. *V_ij_* is usually expressed as a linear function:

4.2 $$V_{ij}=\beta_{0}+\beta_{1}X_{1}+\beta_{2}X_{2}+\cdots +\beta_{i}X_{ij},$$

where *β*_0_, *β*_1_, *β*_2_, … , *β_i_* are the parameters to be estimated; *X*_1_, *X*_2_, …, *X_ij_* are the significant factors influencing pedestrians to choose strategy *j*.

Assuming the random terms independently follow the Gumbel distribution, then the probability that the decision maker *i* choose strategy *j* is

4.3 $$P_{ij}=\frac{e^{V_{ij}}}{\sum_{\;j=1}^{J}e^{V_{ij}}}.$$

#### 4.1.1. Definition of the multi-nomial logit model

The definition of the MNL model variables using characteristic parameters defined in §3.2 is shown in table 1 and table 7. Due to the reason that both *D* and *θ* are mainly accounted for by the indicator of deviation from the shortest path to the destination, only one of them has been kept in the model. In order to test the significance of these variables to the dependent variable, the other eight variables are firstly put into the MNL model; these variables are tested according to the significance. Results of using all the eight variables are shown in table 8, and the results after the MNL Model analysis are shown in table 9, where *X*_4_ and *X*_6_ are discarded because they are not significant to the dependent variable according to the test with a significance level of 0.05. The MNL model analysis results in similar pseudo *R* squares for the eight-variable model (0.536) and the six-variable model (0.533), indicating that using six variables should be an appropriate substitution to eight.

**Table 7.** Definition of the potential independent variables of the MNL model and the corresponding characteristic parameters of CA.

potential independent variables of MNL model	*X*_1_	*X*_2_	*X*_3_	*X*_4_	*X*_5_	*X*_6_	*X*_7_	*X*_8_
characteristic parameters of CA	*v_i_*_(*t*)_	*N*	$\bar{d}$	*d*_min_	$\bar{\beta }$	*β*_min_	*D*	*α*

**Table 8.** Results of the MNL model when all the eight parameters are used as inputs.

var.	acceleration		deceleration		detour		stop	
	*β*	*p*	*β*	*p*	*β*	*p*	*β*	*p*
*X*_1_	9.794	0.000	−35.683	0.000	2.958	0.243	−119.693	0.099
*X*_2_	−2.214	0.628	17.223	0.007	2.494	0.562	41.414	0.445
*X*_3_	−7.267	0.190	16.849	0.046	−4.261	0.418	22.962	0.778
*X*_4_	5.628	0.344	−10.569	0.229	2.539	0.654	−9.623	0.904
*X*_5_	4.298	0.017	−7.574	0.070	5.023	0.003	−4.229	0.921
*X*_6_	1.153	0.383	−0.794	0.652	1.410	0.265	5.700	0.747
*X*_7_	24.018	0.000	1.527	0.748	19.301	0.000	−6.173	0.917
*X*_8_	−21.813	0.000	−1.647	0.571	−16.707	0.000	13.469	0.767
const.	−7.401	0.001	15.080	0.000	−3.700	0.055	13.801	0.779

Therefore, the fixed-term part of pedestrian decision-making utility in the MNL model can be expressed as $\;V_{n}=\beta_{1}\cdot X_{1}+\beta_{2}\cdot X_{2}+\beta_{3}\cdot X_{3}+\beta_{5}\cdot X_{5}+\beta_{7}\cdot X_{7}+\beta_{8}\cdot X_{8}$. And then the unknown parameters *β*_1_, *β*_2_, *β*_3_, *β*_5_, *β*_7_, *β*_8_ can be estimated through the MNL model by using the maximum-likelihood estimation.

#### 4.1.2. Results of model estimation and validation

In order to understand and predict the influences of the characteristic parameters on pedestrians' CA strategies in the complex interweaving area, the aforesaid MNL model of CA strategy choice has been estimated based on the maximum-likelihood estimation method.

For the probability density function of the decision maker *i* choose strategy *j*, *P_ij_*(*X*|*β*), the likelihood function of variable *X* = {*x*_1_, *x*_2_, … ,*x_n_*} for *β* in the sample data is as follows:

4.4 $$L(\beta )=\overset{I}{\underset{i=1}{\prod }}\overset{J}{\underset{\;j=1}{\prod }}\;p_{ij}^{\;f_{ij}},$$

where *I* is the total number of samples and *J* is the total number of strategies. *f_ij_* is a binary variable introduced to formulate the probability that the decision maker *i* chooses strategy *j*: *f_ij_* = 1 when decision maker *i* chooses strategy *j*, otherwise, *f_ij_* = 0.

Logarithm is usually taken on both sides of the above equation for the convenience of parameter estimation.

4.5 $$H(\beta )=lnL(\beta )=\overset{I}{\underset{i=1}{\sum }}\overset{J}{\underset{\;j=1}{\sum }}f_{ij}ln(p_{ij}(X|\beta )).$$

Then, parameters *β* can be estimated by solving the maximum problem of the likelihood function.

The empirical data of the aforesaid six characteristic parameters extracted from the three groups of interweaving pedestrian flow experiment were used to estimate the model, and the data of pedestrians without CA strategies were used as the comparison group. The model estimation results of the three groups of experiments are shown in table 9.

**Table 9.** Results of model estimation and validation.

alternative choices (CA strategies)	variables	*β*	s.e.	*p*	pseudo *R*^2^	accuracy of validation
acceleration	*X*_1_	6.9167	2.556	0.007	0.533	0.806
	*X*_2_	0.3398	2.352	0.885		
	*X*_3_	−1.6408	1.5550	0.290		
	*X*_5_	4.0246	1.714	0.019		
	*X*_7_	26.3516	5.836	0.000		
	*X*_8_	−23.7618	5.251	0.000		
	const.	−4.7051	1.937	0.015		
deceleration	*X*_1_	−42.5442	9.913	0.000		
	*X*_2_	11.5395	4.086	0.005		
	*X*_3_	9.7155	3.915	0.013		
	*X*_5_	−10.6753	4.919	0.030		
	*X*_7_	3.3908	4.619	0.463		
	*X*_8_	−3.4148	2.926	0.243		
	const.	18.3549	4.448	0.000		
detour	*X*_1_	0.2657	2.459	0.914		
	*X*_2_	3.7579	2.213	0.089		
	*X*_3_	−1.0143	1.518	0.504		
	*X*_5_	4.7275	1.642	0.004		
	*X*_7_	20.9036	4.713	0.000		
	*X*_8_	−17.8010	3.752	0.000		
	const.	−1.5853	1.833	0.387		
stop	*X*_1_	−82.7487	21.364	0.000		
	*X*_2_	22.1075	9.178	0.016		
	*X*_3_	15.4884	9.854	0.116		
	*X*_55_	−14.1658	13.410	0.291		
	*X*_7_	6.1216	17.422	0.725		
	*X*_8_	−0.5632	15.170	0.970		
	const.	20.7487	13.334	0.120		

**Figure 16.**
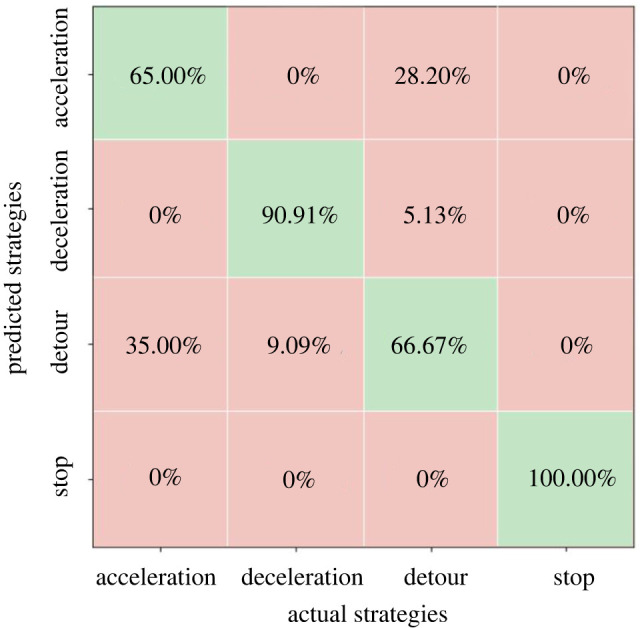
Percentages of predicted number of choices in each category by the MNL model to the real number of choices in the corresponding category, cells in green colour represent accuracy of the model in the corresponding category.

To validate the accuracy of the MNL model, the model-predicted CA strategy choices are compared with real choices observed in the experiment. Figure 16 shows the percentages of model-predicted number of choices in each category to the real number of choices observed. It can be seen that the accuracy for C and S strategy categories can reach 90.9% and 100%, respectively, but the accuracy for A and T strategy categories is not very satisfactory, yielding an overall accuracy of the MNL model prediction 80.6%. This is partially because some of the SPs may accelerate during detour behaviours, but this situation is categorized as detour strategy in this study. Another potential drawback of the MNL model is that the modelling mechanism does allow ‘time-series’ mode of model, that is, the inputs can only take the values of these variables at one time point, and this study uses the averages of these variables in the past 10 frames, but not the original time-series data in the 10 frames. For this reason, the next section of this paper tries to use a LSTM model to replace the MNL model and compares these two types of models.

### 4.2. The long-short-term-memory model for collision avoidance strategy choice

As mentioned above, using the time-series data during a small time period, from *t* − Δ*t* to *t*, to predict pedestrians' decisions at time *t* + 1 could be a promising way to model the nature of human decision-making behaviours during CAs. This paper adopts the LSTM model to test this assumption based on the same set of data and dependent variables used in the aforesaid MNL model. The only difference is that instead of using the averages, the data of the past 10 frames of each variable are used as inputs directly.

**Figure 17.**
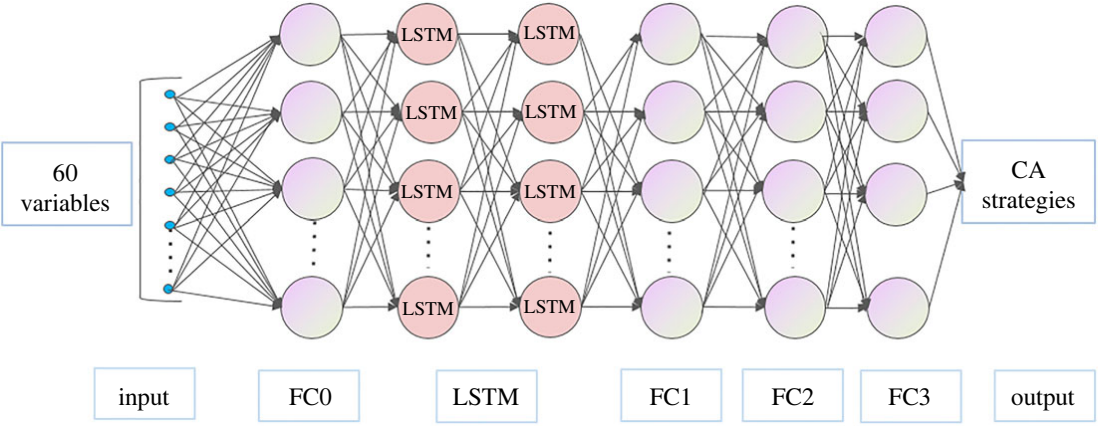
Topological network structure of the LSTM model.

The LSTM model is built based on Pytorch. The model consists two LSTM cells with an overall topological network structure as shown in figure 17. The details of the connections between layers are presented in table 10. The model uses the time-series data in the past Δ*t* = 10 frames (taking the moment of the decision making as time *t*) of the six variables in table 9 as the inputs. Therefore, totally 60 input parameters are introduced into the input layer to represent the current pedestrian movement status (table 11). The values of these variables are normalized to avoid the influence of different units of these variables. The output parameter is the pedestrian's choice of one of the CA strategies.

**Table 10.** Details of the structure of the LSTM model.

layer name	details
full connected layer (FC0)	in_features = 6 × 10, out_features = 256
LSTM layers	input_size = 256, hidden_size = 256, dropout = 0.2, num_layers = 2
full connected layer (FC1)	in_features = 256, out_features = 128
full connected layer (FC2)	in_features = 128, out_features = 64
full connected layer (FC3)	in_features = 64, out_features = 4
softmax layer (out)	in_features = 4, out_features = 1

**Table 11.** Inputs of the proposed LSTM.

index of input variables	data used for these variables	unit
1,7,13,19,25,31,37,43,49,55	values of *X*_1_ in the past 10 frames	m s^−1^
2,8,14,20,26,32,38,44,50,56	values of *X*_2_ in the past 10 frames	—
3,9,15,21,27,33,39,45,51,57	values of *X*_3_ in the past 10 frames	degree
4,10,16,22,28,34,40,46,52,58	values of *X*_5_ in the past 10 frames	degree
5,11,17,23,29,35,41,47,53,59	values of *X*_7_ in the past 10 frames	m
6,12,18,24,30,36,42,48,54,60	values of *X*_8_ in the past 10 frames	degree

**Figure 18.**
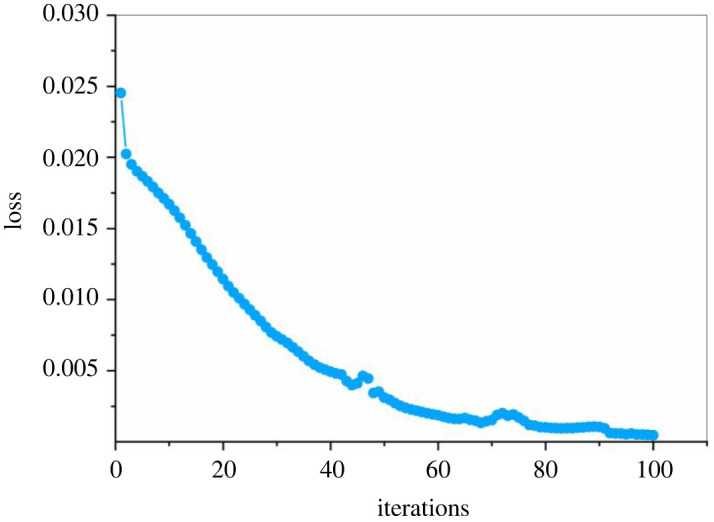
The trend of the loss during the training phase of the LSTM model.

The hyper-parameters of the LSTM model used in this study is shown in table 12, including the learning rate, batch size, number of iterations and the choice of the optimizer, loss function and activation function, and the trend of loss during model training is shown in the figure 18.

**Table 12.** Parameter setting of the LSTM model.

parameters	value
learning rate	0.0001
batch size	45
optimizer	Adam
loss function	CrossEntropyLoss
activation function	Relu
number of iterations	100

**Figure 19.**
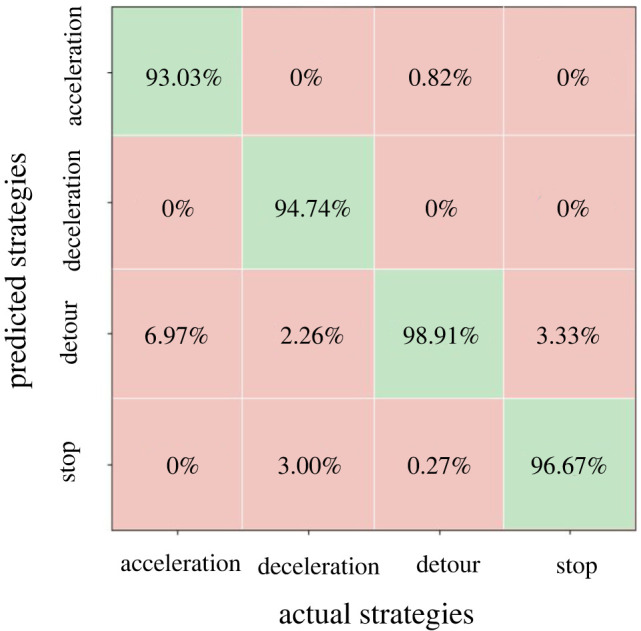
Percentages of predicted number of choices in each category by the LSTM model to the real number of choices in the corresponding category, cells in green colour represent accuracy of the model in the corresponding category.

The LSTM model is trained based on the above settings using 70% of the data; the remaining 30% of the data are used for validation purpose. As shown in figure 19, the accuracy of LSTM model for each type of CA strategy are all higher than 93%, with an overall accuracy of 95.83%. The validation results show that the LSTM model outperforms the MNL model a lot and is good enough for predicting pedestrians' CA strategy choices in interweaving pedestrian flows.

## 5. Conclusion

Considering the insufficiency of research on the behavioural characteristics of pedestrians adopting different CA strategies to avoid other pedestrians in interweaving pedestrian flows, this study carries out a series of controlled interweaving pedestrian flow experiments with different densities to investigate the CA behaviours, especially CA strategy choices. Four types of CA strategies, including ‘Acceleration’ (A), ‘deCeleration’ (C), ‘deTour’ (T) and ‘Stop’ (S), are manually identified in these experiments. Nine characteristic parameters of pedestrian CA behaviours based on the trajectory data are defined to explore the characteristics of CAs as well as the interrelationships between CA strategies and the locomotion conditions.

Three groups of experiments, with low, medium and high pedestrian flow density levels, are included in the experiment. Pedestrian movement trajectory data are extracted using the PeTrack software, and the data of the nine characteristic parameters are calculated based on the trajectory data. Each frame of trajectory data was given a label denoting whether the pedestrian is at NWS or at CAS and which types of CA strategies have been chosen.

It can be found from the experimental data that as the density level increases, pedestrians need more time to walk across the interweaving area, and velocities slow down as the trajectories deviate further from the shortest path; pedestrians have to carry out more times of CAs, and the detour, deceleration or stop CA strategies appear more often due to the closer interaction distances and more aggressive movement direction conflicts during CAs.

Heterogeneous pedestrian CA behaviours can be identified in the experiment. Pedestrians with higher normal walking velocities tend to accelerate or detour more often. Although the averages of minimum distance and relative movement direction to other pedestrians during CAs range widely from 0.5 to 1.7 m and 92 to 110 degrees, respectively, the thresholds of these parameters for different individuals vary.

Totally 258 CASs are segmented from the trajectory data. The time intervals between two consecutive CASs widely distribute in the range of zero frame to over 100 frames, but the mean is 26.87 frames, and most of the interval lengths (over 70%) are longer than 10 frames. The time lengths of the CASs range from 0.5 to 3.5 s, but most of them are within the range of (0.5, 2.5) s, with the mean of 1.3–1.8 s. No obvious differences could be identified either between different groups of experiments or different types of CASs. The trajectory lengths of CASs of A, C and T strategies range from 0.5 to 3.5 m, with the mean of 1.35–2.22 m.

MIC between each pair of characteristic parameters show that medium or high level of correlations (MIC greater than 0.4) exist between most of the parameter pairs. Analyses of the relationships between CA strategy choices and the characteristic parameters reveal that the average values of these parameters are different during the executions of different CA strategies, implying possible mapping relationships between these parameters and the behavioural choices.

A MNL model and a LSTM model are then established, respectively, for predicting pedestrians' choices of CA strategies using the selected characteristic parameters as inputs. The modelling results prove that the LSTM model makes better use of the time-series data in the past short time period, from *t* − Δ*t* to *t*, (Δ*t* = 0.4 s in this study) to predict the CA strategy choices at time *t* + 1 and lead to higher prediction accuracy (with overall accuracy of 80.6%) than the MNL model (with overall accuracy of 95.83%), especially for the A and T strategies.

These findings of this study are useful for understanding the pedestrian flow interweaving movement mechanisms from the individual movement and interaction perspectives, especially about the patterns and the quantitative characteristics of CA locomotion behaviours and the CA strategy choices. These qualitative or quantitative results may support future microscopic pedestrian flow modelling and parameter calibration studies, and can also provide a theoretical basis for the design of pedestrian facilities and emergency evacuation in crowded places.

Limitations of the current study include: (i) only three density levels and pedestrians with the age of around 20 in China were considered in the experiment leading to incomplete estimation of pedestrian movement characteristics, which means transferring the results in this paper to other density levels or other types of pedestrian groups cannot be guaranteed; (ii) in order to guarantee reasonable complexity of data acquisition tasks and sample size of CA strategy data, this paper does not distinguish different types of mixed CA strategies, such as detour while accelerating or decelerating, but this should be considered if there are enough data to cover each more detailed type of CA strategies in future; (iii) this study has focused on the choice of CA strategies under a given situation, but was not designed to answer the question that under what kind of situations pedestrians would have to make decisions of CA, i.e. the deeper and more general relationships between ‘states' of pedestrians' walking environment and the ‘actions’ of pedestrians still need further studies.

Despite the above limitations, this paper proves that using time-series data for predicting pedestrian behavioural choices at the next time step by machine learning models such as LSTM is a meaningful attempt and might be a promising direction. Adopting similar techniques to develop robust data-drive microscopic pedestrian flow simulation models by considering the consistency of pedestrian behaviours along the time line could be a future work.

## Data Availability

The trajectory data used in this paper are available from the Dryad Digital Repository (https://doi.org/10.5061/dryad.c2fqz619v) [44].
